# Gait-Based Implicit Authentication Using Edge Computing and Deep Learning for Mobile Devices

**DOI:** 10.3390/s21134592

**Published:** 2021-07-05

**Authors:** Xin Zeng, Xiaomei Zhang, Shuqun Yang, Zhicai Shi, Chihung Chi

**Affiliations:** 1 School of Electronic and Electrical Engineering, Shanghai University of Engineering Science, Shanghai 201620, China; xinzeng@sues.edu.cn (X.Z.); shqyang@sues.edu.cn (S.Y.); shizhicai@sues.edu.cn (Z.S.); 2 Commonwealth Scientific and Industrial Research Organization (CSIRO), Sandy Bay 7005, Australia; chihung.chi@csiro.au

**Keywords:** implicit authentication, gait recognition, convolutional neural network, LSTM, edge computing

## Abstract

Implicit authentication mechanisms are expected to prevent security and privacy threats for mobile devices using behavior modeling. However, recently, researchers have demonstrated that the performance of behavioral biometrics is insufficiently accurate. Furthermore, the unique characteristics of mobile devices, such as limited storage and energy, make it subject to constrained capacity of data collection and processing. In this paper, we propose an implicit authentication architecture based on edge computing, coined Edge computing-based mobile Device Implicit Authentication (EDIA), which exploits edge-based gait biometric identification using a deep learning model to authenticate users. The gait data captured by a device’s accelerometer and gyroscope sensors is utilized as the input of our optimized model, which consists of a CNN and a LSTM in tandem. Especially, we deal with extracting the features of gait signal in a two-dimensional domain through converting the original signal into an image, and then input it into our network. In addition, to reduce computation overhead of mobile devices, the model for implicit authentication is generated on the cloud server, and the user authentication process also takes place on the edge devices. We evaluate the performance of EDIA under different scenarios where the results show that i) we achieve a true positive rate of 97.77% and also a 2% false positive rate; and ii) EDIA still reaches high accuracy with limited dataset size.

## 1. Introduction

Mobile devices, including smartphones and tablets, play important roles in our daily lives. With the growth in their use, people increasingly store ample private and sensitive information such as photos and emails on mobile devices. Therefore, it is essential to prevent private data from threats caused by unauthorized access [1,2,3]. Traditional access control methods employ one-time authentication mechanisms, e.g., passcodes, PINs, face recognition and fingerprints, which is asked when users try to start-up their mobile devices. However, these authentication mechanisms are easy to crack by guessing, smudge attacks and static photos [4]. Moreover, users is required to present these credentials only for initial login. Unauthorized users may illegally access to devices if the authentication system is not constantly on guard against intruders after initial login.

To cope with these challenges, researchers develop implicit authentication to continuously monitor user identification in runtime via behavioral biometrics [5,6]. There are plenty of mobile implicit authentication methods in the literature, e.g., touch dynamics-based approaches, behavior-based profiling, and gait dynamics based approaches [4]. Touch dynamics-based authentication methods utilize the way users interact with the touch screen of their mobile devices. Behavior-based profiling methods identify users in terms of the tasks and services they prefer to use. In practice, these two types of authentication make users be constrained to perform some appointed tasks or wear some devices for collecting the desired data. Rather than obtrusive data collection process, gait dynamics based schemes need less user interaction and no additional hardware required.

More recently, gait biometrics has received academic attention in implicit authentication techniques for mobile devices [7]. Usually these schemes do not achieve a Training Accuracy Rate (TAR) higher than 95% with EER from 5% to 10%. Further works need be devoted to improving accuracy. Some new developments in behavior recognition via deep learning can be applied in the field of implicit authentication [8,9]. Rather than other machine learning models, deep learning emancipate us from the hard manual work of hand-crafted feature extraction. In this paper, user authentication has been proposed through the use of a hybrid approach which consists of a Convolutional Neural Networks model and Long Short-term Memory Recurrent Neural Network (CNN-LSTM). Compared to the work only based on a CNN model, our hybrid approach is able to improve the accuracy of the result by almost 3%.

Although deep learning is successful in image and speech classification, its application for non-image/speech data, including human behavior recognition using raw sensor data, brings new challenges. As there are abundant noises in raw data, this means behavior recognition may lead to lots of errors during deep learning based authentication. We design a data process method of translating the gait signal in two-dimension domain, inspired by previous state of the art [10] which have been applied to fault detection and diagnosis using features of vibration signal. In the process of transforming data to an image, all the data of images are preserved. Meanwhile, the noise in the data is transformed into the gray and brightness information of the image, which reduces the impact of noise on the classification results. This are intended to achieve the purpose of noise reduction initially, and so to improve the accuracy of our authentication model.

In this paper, we propose an efficient implicit authentication system named Edge computing-based mobile Device Implicit Authentication (EDIA), which uses the gait biometric traits generated by users and captured by build-in sensors. In EDIA, edge computing paradigm alleviates the computation burden of mobile devices through offloading computing task to the nearest edge node, while computing paradigm is leveraged to generate the authentication model which could achieve real-time user authentication. We introduce an image conversion method which converts the raw gait signal to 2D format as the input of deep learning model and further promote the performance of our model. User authentication is employed through a hybrid method including CNN and LSTM, where CNN is utilized as a feature extractor and the LSTM is used as a classifier. Various experiments and tests are carried out to show the effectiveness and robustness of EDIA in different scenarios. Furthermore, our authentication method also achieves high authentication accuracy with small data sets, indicating that our model is suitable for mobile devices with insufficient battery and computing power. Although gait as a biometric trait has been used in user authentication, most works focus on the gait signal’s frequencies and magnitude, which are dealing with one dimension domain. Few studies looked into multi dimension domains. Specifically, to the best of our knowledge, no other work has applied gait signal-based image (two dimensions) conversion pre-processing on deep learning methods for user authentication.

The highlights and contributions of this paper can be summarized as follows:

- We propose an edge computing-based implicit authentication architecture, EDIA, which is designed to attain high efficiency and computing resources optimization based on the edge computing paradigm;
- We develop a hybrid model, based on concatenation of CNN and LSTM accommodated to the optimized process of gait data from build-in sensors.
- We present a data preprocessing method, which extracts the features of a gait signal in two-dimension domain by converting the signal into an image. By this way, the influence of noise on classification results is reduced and then the authentication accuracy can be improved.
- We implement and evaluate the authentication performance of EDIA in different situations on a dataset which is collected by Matteo Gadaleta et al [11]. The experiment results show that EDIA achieve an accuracy of 97.77% with the 2% false positive rate and demonstrate the effectiveness and robustness of EDIA.

## 2. Related Work

To cope with the drawbacks of traditional authentication methods such as passwords, researchers turn their attention to behavioral-based approaches in recent years. Continuous implicit authentication techniques based on behavioral features have been being studied and developed rapidly on mobile devices such as tablets and cell phones. Basically, they detect user’s identity based on the physiological and behavioral biometrics information from sensors that are built into the mobile device. This includes gyroscopes, pressure sensors, touch screens, orientation sensors and accelerometers.

Touch screen is the main interaction interface between users and smart mobile devices nowadays. This is one of the most popular ways to gather behavior information because they can be obtained without the help of other hardware and can be analyzed in real-time. Since differences in user’s touch screen trajectory, speed and click position can produce different pressures on the screen, these behavioral features of a user can be recognized distinctly for implicit authentication. As an example, Frank et al. [12] proposed an implicit authentication framework based on the way users interact with the touch screen. They use a set of 30 behavioral touch features that can be extracted from the touch screen logs to authenticate users. After data processing and feature analysis, they utilized both kernel support vector machine (SVM) and k-nearest-neighbors (KNN) to evaluate the features on a dataset consisting of 41 users’ touch gestures. The experimental results show that an EER of 0% to 4% can be achieved in different application scenarios.

Behavior-based authentication methods can also authenticate users by analyzing applications and services that people use on their phones. In these systems, user profiles, which are generated through monitoring user’s activity on the phone over a period of time, are compared against the current activity profile to identify users. A significant deviation means that an intrusion may have been detected. Recently, some studies apply this technique to implicit authentication [13,14,15]. In these studies, application-level as well as application-specific characteristics such as cell phone ID, data, time and number of calls, call time, and application usage time are used to authenticate the user. Li et al. [13] proposed a method based on behaviors of using telephony, text messaging, and general application usage as features. They achieved EERs of 5.4%, 2.2%, and 13.5% on the MIT Reality dataset [16].

Keyboard input is another common interaction method for mobile devices. People are likely to have their own keystroke input characteristics, e.g., keystroke delay, strength, duration, and keystroke location, which can be analyzed during the use of keyboard for identification. Keystroke authentication is further divided into fixed text and free text studies. Lee et al. [17] studied the dynamic features of user keystrokes and proposed a parametric model approach that can select the most distinguishing features for each user with a false rejection rate of 11%. However, keystroke behavior gradually decreases on most mobile devices since there is no virtual keyboard on lots of wearable devices [18]. Therefore, implicit authentication based on this behavioral feature is not applicable to popular mobile devices such as smart glasses. Hand waving patterns of a person are yet another unique, stable and distinguishable feature. Han et al. [19] proposed a system named OpenSesame which exploits users’ hand waving patterns and leverages four fine-grained and statistic features of hand waving. In addition, they used the support vector machine (SVM) as classifier. The problems and limitations of the above implicit authentication methods are described in Table 1.

With the increasing number of sensors in standard mobile phones, gait features are beginning to garner people’s attention for authentication. Not only are the gait features stable, distinguishable, and easy to collect, but the time that people have their phone with them is also increasing. Furthermore, the number of gait features available is plenty. A gait-based implicit authentication method identifies users by the way they walk. Gait signal data, such as those from accelerometer and gyroscope sensors, can be easily obtained through the phone’s built-in sensors. With these continuous data streams being collected, discriminative gait features can be extracted and then be trained by the classifier for authentication. A number of methods have been proposed to perform gait-based implicit authentication on mobile devices. These methods differ mostly in the ways features and extracted from the raw data or the algorithm used for authentication.

In [20], Mantyjarvi et al. used the methods based on correlation, frequency domain analysis, and data distribution statics, while Thang et al. [21] and Muaaz et al. [22] implemented the dynamic time warping (DTW) method. Instead of using the gait cycles for extracting features, Nickel et al. [23] used hidden Markov models (HMMs) for gait recognition. The methods mentioned above require feature selection and extraction before user authentication can be performed, and these processes are usually tedious and complex. Zhong et al. [24] proposed a sensor direction-invariant gait representation method called gait dynamic images (GIDs). The basic idea is to capture the three-dimensional time series using a triaxial accelerometer, and GDI is expressed as the cosine similarity of the motion measurement at time *t* with the lag time signal. Damaševicius et al. [25] used random projections to reduce feature dimensionality to two, followed by computing the Jaccard distance between two probability distributed functions of the derived features for positive identification. Kašys et al. [26] performed user identity verification using linear Support Vector Machine (SVM) classifier on his/her walking activity data captured by the mobile phone. Xu et al. [27] presented Gait-watch, a context-aware authentication system based on gait feature recognition and extraction under various walking activities. Abo El-Soud et al. [28] used filter and wrapper approaches for feature selection of gait data and random forest classifier for the authentication. Papavasileiou et al. [29] proposed GaitCode, a continuous authentication mechanism based on multimodal gait-based sensor data. They used a network of auto-encoders with fusion for feature extraction and SVM for classification.

Table 2 summarizes the performance of gait-based implicit authentication methods on various datasets. One common weakness shared among these methods is the requirements of data pre-processing operations and manual feature screening processes for the collected gait signals before authentication. They are not only tedious and complicated, but also affect the subsequent authentication accuracy if the features are not selected properly.

The field of deep learning [30] has demonstrated its effectives in areas related to gait recognition. This includes action recognition [31], video classification [32] and face recognition [33]. However, the training of deep learning models requires a large amount of data, and it is very difficult to collect a large amount of gait data because of the battery and processing power limitations of mobile devices. Compared to traditional machine learning methods, deep learning methods are less applied to gait-based implicit authentication problems. Gadaleta et al. [11] proposed a user authentication framework from smartphone-acquired motion signals named IDNet. IDNet exploits CNN as universal feature extractors, which still uses one-class SVM as a classifier. They directly use the original gait signal as the input to the convolutional neural network. This method is not appropriate for CNN because CNN is not good at processing one-dimensional signals. Giorgi et al. [25] described a user authentication framework, exploiting inertial sensors and making use of Recurrent Neural Network for deep-learning based classification. However, the difference in results for known identities and unknown identities is quite significant.

To face above challenges, in this paper, we choose gait features as inputs of a CNN-LSTM model to identify mobile device users. Before the gait data collected by the device is fed into the network, we convert it into a two-dimensional image. Our authentication system not only inherits the advantages of deep learning, but also achieves initial noise reduction, all of which can promote the accuracy of authentication.

## 3. The Methodology

**Figure 1:**
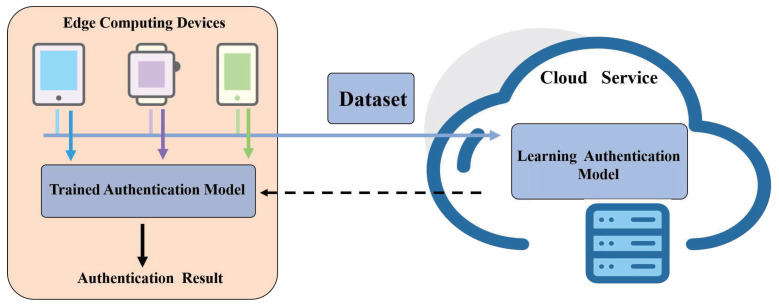
The framework of authentication.

In this section, we propose an edge computing-based mechanism named EDIA to authenticate mobile device users. The architecture of EDIA is shown in Figure 1. EDIA includes a model training module deployed in the cloud and a user authentication module deployed on edge devices (e.g., smartphones, smart tablets, and other mobile devices). Deploying the training module with the cloud can provide powerful computing power for model training to speed up the training process. When a legitimate user registers for the first time, the system continuously collects the user’s gait information through the smart mobile device at the edge and sends it to the cloud for generating authentication feature vectors. We deployed the data collection and model generation modules in a trusted cloud-based server. To protect the security of user information, all user data were anonymized and the cloud model has access to the data but not to the user information of the gait data. The model generation module was trained with the gait information of legitimate users and the information of other users to generate user authentication models. After training, the user authentication model is downloaded to edge devices such as cellphones to ensure real-time user authentication. The training module in the cloud is not involved in the user authentication process, and only when the model on the mobile device detects a deviation in the gait behavior of the legitimate user, the training module will re-collect data for training, a process that occurs automatically in the cloud. Thus EDIA does not require the mobile device at the edge to be in constant communication with the cloud, further reducing the risk of intrusion.

**Figure 2:**
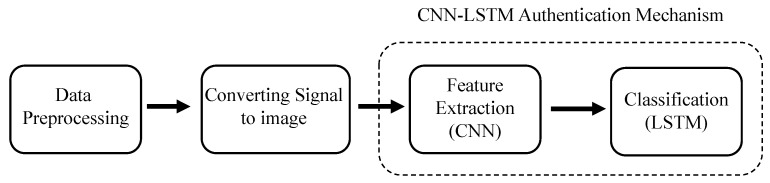
The framework of authentication.

The process of implicit authentication of mobile device users based on gait data is shown in Figure 2, which consists of three basic stages: data preprocessing, convert signal to image, and CNN-LSTM authentication mechanism. The authentication mechanism is composed of two different networks, CNN is responsible for feature extraction, whereas LSTM acts as a classifier.

### 3.1. Data Preprocessing

The dataset used in this paper were proposed by Gadaleta et al. [11]. The dataset is composed of 50 people who walked in their natural state. The data were monitored and captured by the smartphones with the inertial sensors, which were put in the right front of the users’ trousers. There are problems of missing data and high frequency signal interference in the process of collecting gait signals from the built-in sensors of cell phones, so the data needs to be processed to facilitate the subsequent user authentication process.

#### 3.1.1. Filter and Gait Cycle Extraction

**Figure 3:**
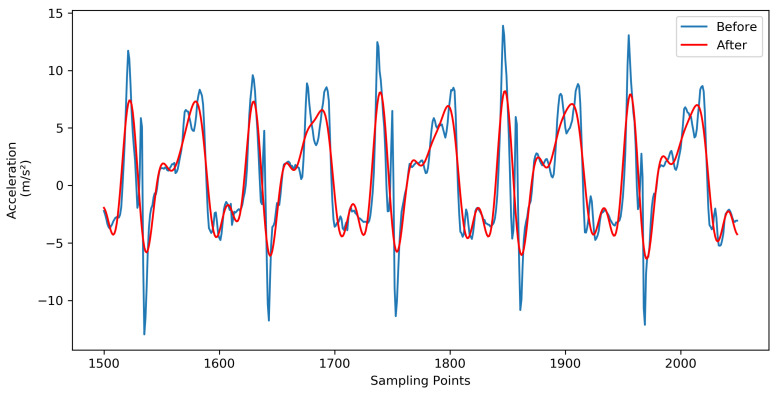
Comparison of gait signal before and after filtering.

The gait signal generated by a person while walking is a low frequency signal, and the cell phone sensor will receive interference from high-frequency electronic noise and random noise in the process of acquisition, so corresponding means must be taken to remove noise interference to ensure the accuracy of subsequent certification. In this paper, we use the 8th order Butterworth low-pass filter with a cutoff frequency of 5 Hz to remove noise from the signal. Figure 3 shows the comparison of the gait signals before and after filtering.

**Figure 4:**
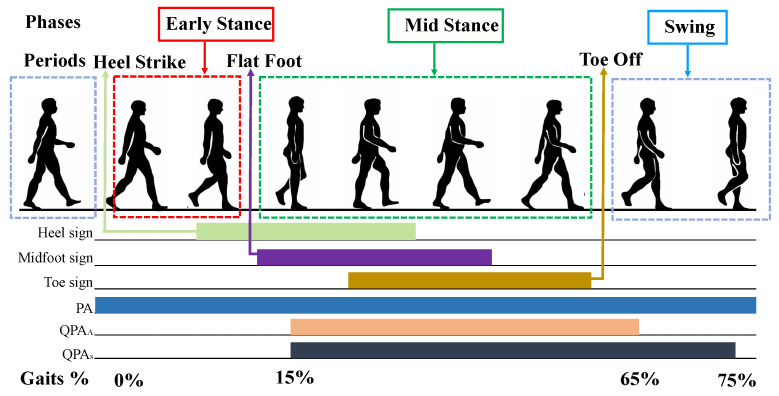
Human gait cycle.

The human walking behavior is a regular quasi-periodic activity, starting from the first contact of the left foot with the ground to the alternation of the right foot until the left foot touches the ground again. Figure 4 shows a diagram of the human gait cycle.

**Figure 5:**
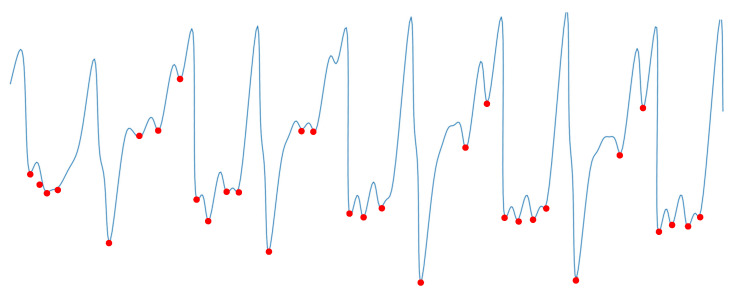
All minimal value points.

In gait-based authentication tasks, the time series data needs to be segmented and the identification is performed for each segmented data (each segmented data is a sample in the identification system). There are two main methods of data segmentation; period-based segmentation and sliding window-based segmentation. With respect to the sequential data of different testers’ distinct movement patterns, the period-based segmentation method divides the data with different lengths. Since the input sample size of the network model in this paper is a fixed value, the period-based segmentation method is difficult to adapt to the recognition model. In contrast, the sliding window-based method can divide fixed data segments, so we adopt the sliding time window-based division method. By the method of minimal value detection, it is found that the length of the sequence in the gait cycle of the user in the dataset was 120–140. The method mainly includes the following steps:

(1) Calculate all minimal values of the input signal by Equation (1)

(1) $$x_{i-1}>x_{i}\&x_{i}<x_{i+1},$$

where $x_{i}$ the sampling point of the current moment, $x_{i-1}$ and $x_{i+1}$ are the sampling points of the previous moment and the next moment, respectively, and the minimal value points of all the gait signals are marked, and the results are shown in Figure 5.

**Figure 6:**
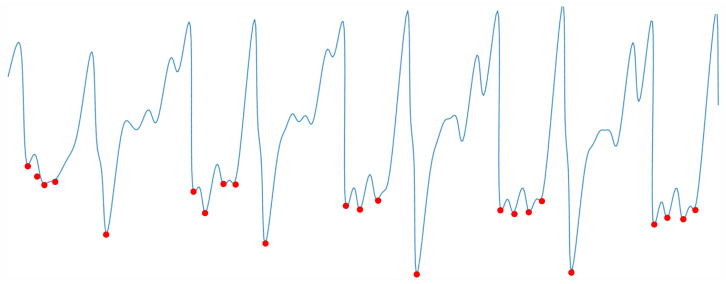
Minimal value after initial screening.

(2) Use the the threshold value obtained from Equation (2) to eliminate some of the pseudo-minority points.

(2) Threshold=mean+0.5std

std dotes the standard deviation of all minima points, mean dotes the mean value of all minima points, result shows in Figure 6.

(3) Use the Equation (3) to obtain the estimated step size of the gait signal.

(3) $$R_{xx}\left(m\right)=\lim_{N\to \infty }\sum_{n=1}^{N}x\left(n\right)x\left(n+m\right)$$

Equation (3) represents the unbiased autocorrelation function of the signal, the autocorrelation function represents the correlation degree of the signal at two different moments, which can reflect the periodicity of the gait signal.

(4) Use the algorithm shown in Algorithm 1 to obtain the gait cycle.

**Figure 7:**
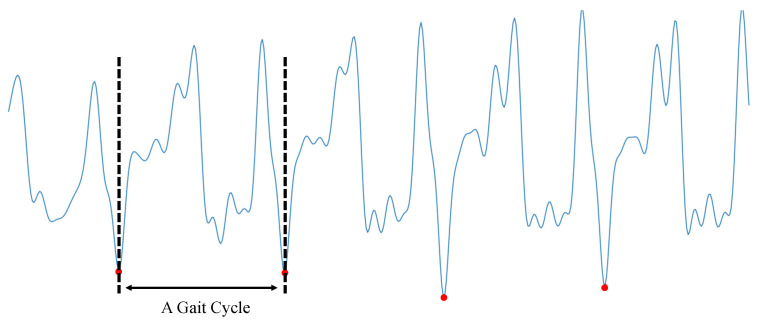
Gait cycle detection.

After the above steps, a gait cycle is obtained as Figure 7 shown.
**Algorithm 1:** Gait cycle detection algorithm. **Input**: Gait acceleration x-axis signal **Output**: Start and end points of each gait cycle **1** Use Equation (1) to obtain all the minimal values of the input signal P1**2** Use Equation (2) to initial screening P1 to obtain P2 after remove the pseudo-minimal values**3** Use Equation (3) to obtain the estimated step size of the gait signal *L*.**4** $\alpha =\frac{1}{4},\beta =\frac{3}{4},\gamma =\frac{1}{6},p=2$**5** **while** p<length(P2) **do****6**    **if** position_of(p)−position_of(p−1)<αL **then****7**    **if** acceleration_of(p)<acceleration_of(p−1) **then****8**       remove(*p*)**9**    **else****10**       remove(p−1**11**    **end****12**    **end****13**    **else if** position_of(p)−position_of(p−1)<βL **then****14**     **if** position_of(p+1)−position_of(p)<γL **then****15**      remove(*p*)**16**     **else****17**      remove(p+1)**18**     **end****19**    **end****20**    p=p+1**21** **end**

#### 3.1.2. Signal-to-Image Convert

In our system, a CNN-LSTM network model is used to authenticate users. While deep networks are not ideal for one-dimensional data, we do some preprocessing, aiming to achieve the purpose of thoroughly using deep networks to extract non-statistical gait features. The gait signals are converted into image data before feeding the data into the network model. Then, the powerful image feature extraction capability of convolutional neural networks is used to extract the non-statistical features of the gait data. The signal-to-image conversion method consists of the following two steps: (1) interception of the gait signal using a sliding window, and (2) conversion of the intercepted gait signal to a grayscale map.

**Figure 8:**
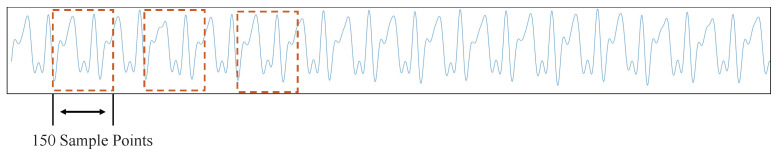
Sliding window.

For one-dimensional gait signals, signal segments need to be intercepted before being converted to images. The signal is intercepted using the sliding window fetch method. After dividing the period of the gait signal by the minimal value detection method (Section 3.1.1), we know that the gait period of human walking is about 120–140. In order to ensure the intercepted data contain a whole gait period, the size of the sliding window is taken as 150. Figure 8 shows the schematic diagram of the sliding window fetching method.

**Figure 9:**
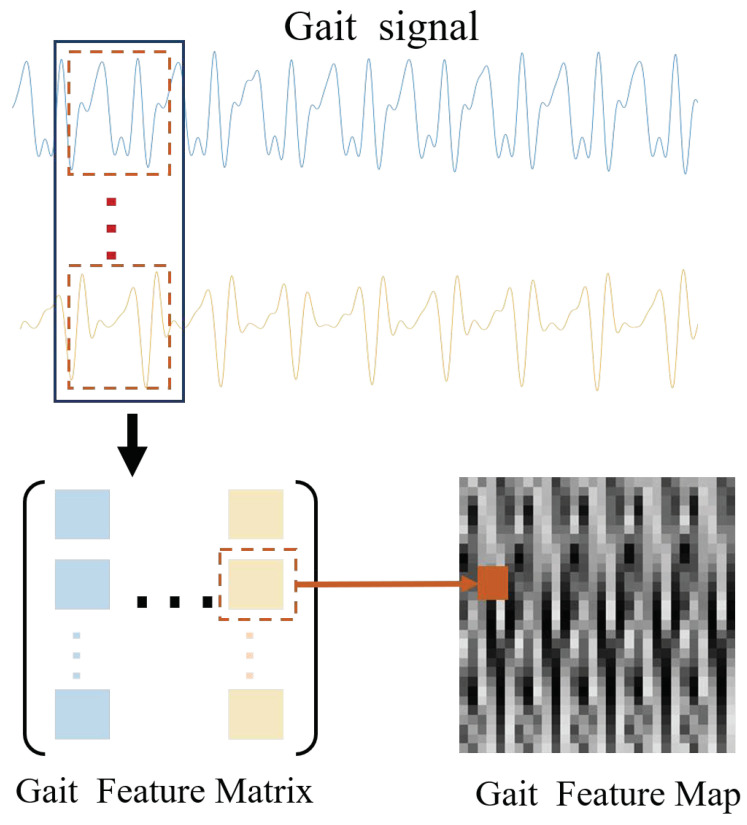
The process of converting.

The process of converting the unfilled signal into an image is shown in Figure 9. In this paper, the data collected by the acceleration sensor and the rotation vector sensor in the dataset are selected for the authentication task. The data collected by each type of sensor includes that in x, y, z axes. Then for each gait signal data, the signal sub-column is intercepted using the sliding window fetching method on the change signal. The sub-columns of the same phase are combined into a gait feature matrix. The specific conversion process is shown in Algorithm 2.

**Figure 10:**
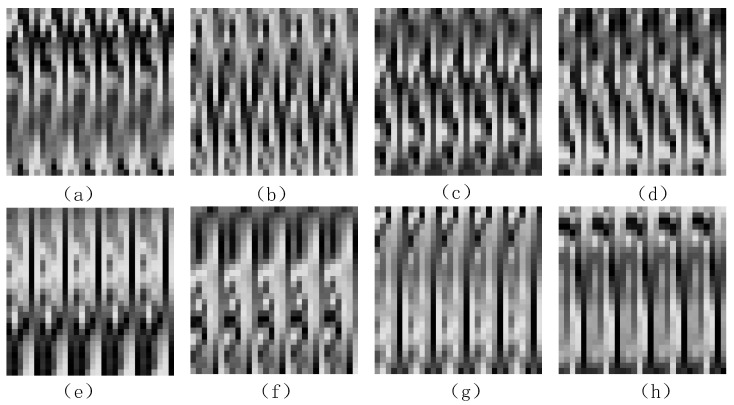
Gait feature image: (**a**–**d**) the feature images of u018; (**e**–**h**) the feature images of u034.

The signal-to-image transformation is completed, and the transformed gait feature map is shown in Figure 10. Figure 10a–d shows the feature images of u018. Figure 10e–h shows the feature images of u034.**Algorithm 2:** Gait feature image generation algorithm. **Input**: Gait signal
 **Output**: Gait feature image **1** **for***column**L**in gait signal***do****2**    M=Max(L)**3**    N=Min(L)**4**    **for** $x_{i}$ *in* ***L*** **do****5**     $x_{map}=\frac{x_{i}-N}{M-N}\times 255$**6**    **end****7**    return L_map**8** **end****9** return signal_map**10** Extraction of feature matrix with sliding window**11** Convert the feature matrix into a feature map

### 3.2. Proposed Architecture

**Figure 11:**
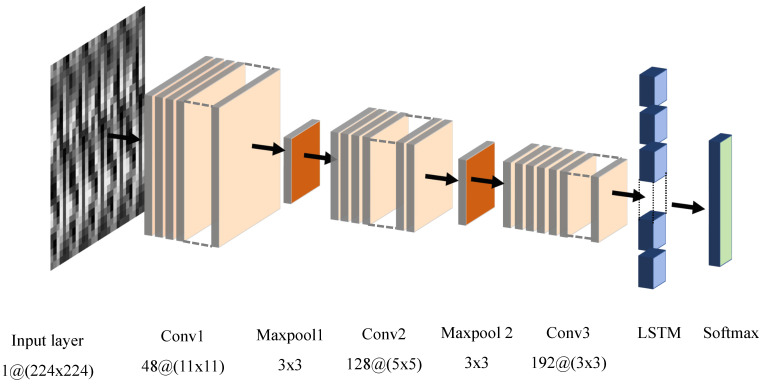
The structure of CNN-LSTM.

The structure of the implicit authentication network based on gait features is shown in Figure 11. For the gait signal, a signal-to-image method is used to convert the one-dimensional gait data into a two-dimension grayscale image. The classification authentication network model is divided into two parts; the CNN network is responsible for extracting the signs, while the LSTM is responsible for classifying the features for authentication.

Convolutional Neural Networks (CNN) are widely-used deep learning models with powerful feature extraction capabilities, which can automatically and efficiently extract features from the input data quickly. Compared with one-dimensional data such as physiological data and financial data, convolutional neural networks are particularly capable of processing two-dimension image data. The inputs in the convolutional layers are connected to the next layers instead of being fully-connected as in traditional neural network models. Sub-regions have the same weights in these input sets, so the inputs of a CNN produce spatially associated outputs. However, in a traditional neural network networks (NN), each input has its own different weight, while the growth of the weights’ number increases the input dimensionality, which make the network more complex. Compared with NN, CNN reduce the weights and the number of connections through weight sharing and downsampling operations. The specific network parameters of CNN are shown in Table 3.

Although CNNs have powerful feature extraction capabilities, they are less effective for some classification/learning tasks where the input is time-dependent such as the gait signal data in this paper. For this type of data, its previous state affects the network’s prediction of subsequent states, so the network is required to not only be ware of the current input but also to remember the previous input. This problem can be solved by the RNN model, which can perform the classification task for each element of the time series. For the present input of RNN, it is not only considered as the current input also as the result of the previous input. The output of RNN at time step *t* is affected by the output of RNN at time step t−1.

**Figure 12:**
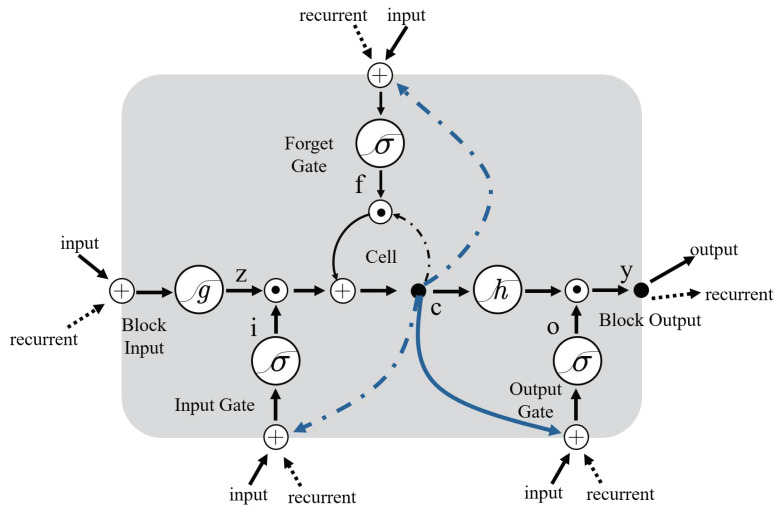
The structure of an LSTM cell.

Theoretically, RNN networks can be used to learn for time series data of arbitrary length. In practice, for large time series, RNN networks suffer from gradient disappearance, which makes it difficult to learn long-range dependencies. To solve this problem, we use a long and short-term memory storage unit as the storage unit of the RNN network, which is the LSTM network. The structure of the LSTM cell is shown in the Figure 12.

LSTM has the ability to remove or add information to cellular states through elaborate structures called ‘gates’. A gate is a method of selectively allowing information to pass through and consists of a sigmoid neural network layer and a pointwise multiplication operation. An LSTM cell has three gates: a forget gate, an input gate and an output gate, to protect and control the state of the cell. Although this paper converts the original gait signal into a two-dimension grayscale image by the data-to-image method, the converted grayscale map is essentially another manifestation of the gait time series. There is still a temporal connection between two adjacent maps, so we use the LSTM plus softmax layer to replace the fully connected layer in the traditional CNN network as the classifier of the authentication network.

## 4. Experiments and Result

### 4.1. Division of the Dataset

The dataset used in this paper contains gait information generated by 50 people. These gait signals are collected by six different brands of cell phones through their four different types of sensors, and two types of information, acceleration sensor and rotation vector sensor, are selected for user authentication in this paper. After preprocessing the data in the dataset with signal image conversion, the number of gait data for each user is shown in Table 4.

**Figure 13:**
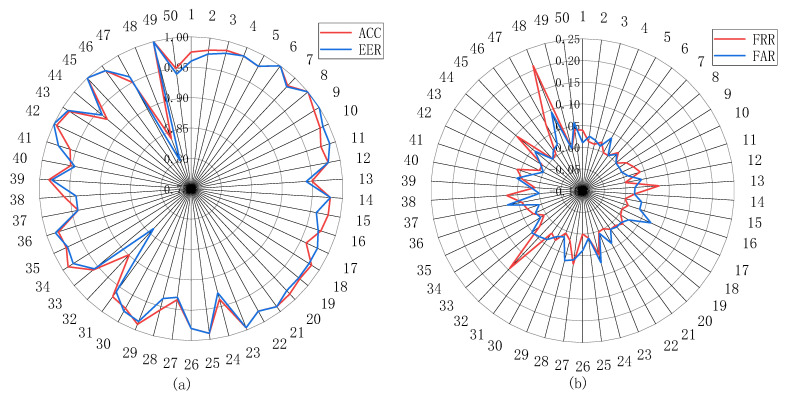
ROC curves, FRR curves and EER curves. (**a**) is the FRR curves and FAR curves (**b**) is the ROC curves and EER curves.

For mobile devices such as smartphones, there is generally only one legal user, so we choose one user as the legal user in this dataset and the rest of the users as illegal users. To validate the performance of the model, we used a leave-one-person-out validation. We separated the dataset into 50 folds, in which the i-th (i = 1, …, N) user, is labeled as legal, and all other users are labeled as illegal. The authentication performance of the model on these 50 datasets is shown in Table 5. The accuracy, ROC, FRR and EER curves is shown in Figure 13. The accuracy distribution statistics of these 50 experiments are shown in Table 6, from which it can be obtained that more than 50% of the accuracy is distributed in the range of 95–97%, and even four times the accuracy reaches 100%, which may be due to the small amount of data for these users.The average accuracy of these 50 experiments was 97.1%

#### Result and Precision Test

We take experiments with different network structures and parameters in order to find the most suitable network model for gait-based implicit authentication. Table 7 shows that the accuracy can achieve by these different networks on the validation set after training and the time required to train these networks. These networks differ mainly in the number of LSTM layers and in the number of LSTM units contained in each layer.

All network architectures achieve positive results in terms of authentication accuracy (above 96%). The increase in the number of LSTM layers increases the authentication accuracy, and all networks containing two layers of LSTM have higher authentication accuracy than networks containing only one layer of LSTM. However, the increased number of layers increase the complexity of the network and lengthen the training time. When the number of LSTM layers is certain, increasing the number of LSTM units in each layer can improve the authentication accuracy of the network; while after it reaches a certain number, increasing the number of LSTM units makes the authentication accuracy decrease. The increase in the number of LSTM units also lengthens the training time, but the impact is not as great as the impact of increasing the number of layers.

**Figure 14:**
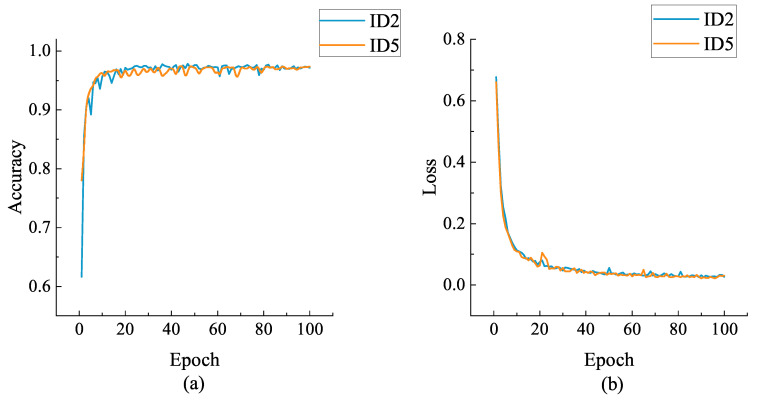
Accuracy curves and loss curves of ID2 and ID5 during training session: (**a**) the accuracy curves; (**b**) the loss curves.

From Table 7, it can be seen that the structure of ID2 and ID5 achieve the best authentication results. To further evaluate the performance of these two networks, we compared their training graphics (as shown in Figure 9). The training graphics show the authentication accuracy of the network in each epoch with respect to the loss. The evolution and convergence trend of the network model can be seen in the Figure 14.

The above figure shows the certified accuracy and loss for each epoch of the two networks during training. Accuracy is the metric whether the label predictions output by the network match the labels of the samples input to the network. The loss is the scalar value of the region minimization, since a lower value means that the value predicted by the model is correct and matches to the true labels of the input samples. The categorical cross-entropy loss function was used in this case. The authentication accuracy curve is correlated with the loss curve, as the authentication accuracy increases, the loss will decrease.

As can be seen from the figure, both structures of the network can converge to a high classification accuracy (above 97%) in a short time and the loss curve can also drop to a very low value quickly, which indicates that both networks have good convergence. However, in comparison, the ID5 network converges slightly faster than the ID2 network, and the training accuracy curve of ID5 converges to a stable value more quickly. In general, the ID5 structure outperforms the ID2 structure because it has an additional LSTM layer, while this makes its training time longer than that of ID2.

### 4.2. Impact of the Number of Datasets on the Authentication Model

For neural network models, the larger the amount of data used for training, the better performance of the final model obtained. However, for implicit authentication of mobile devices, it is difficult to obtain and process large data due to the battery and computing power of mobile devices. Therefore, for the task of implicit authentication of mobile devices based on gait features, we hope that the model requires a smaller amount of data while ensuring the accuracy of authentication.

Ten data sets with different amounts of data are used to train the proposed authentication models in this paper with other conditions held constant. The authentication accuracy achieved by these 10 models on the validation set is shown in Table 8.

**Figure 15:**
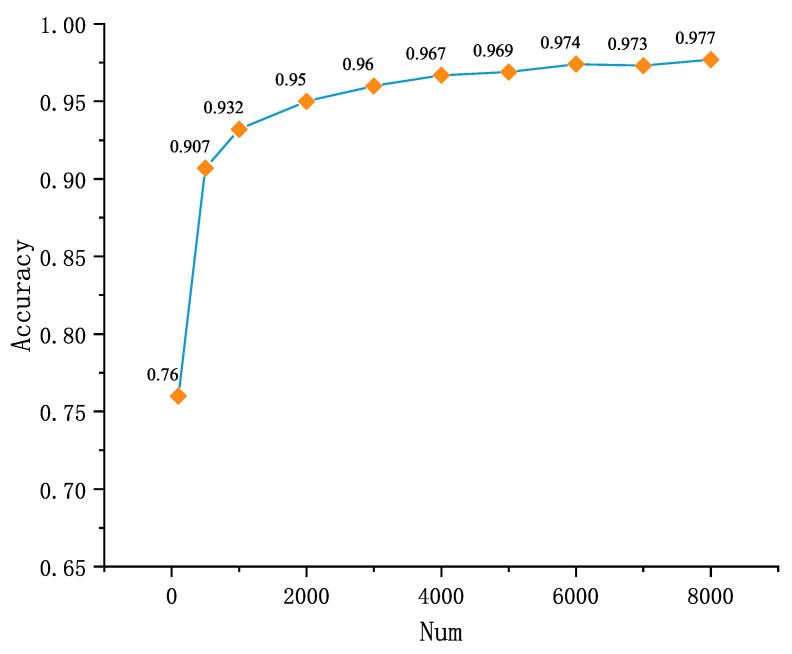
Accuracy achieved on the validation set by the model obtained after training with different number of data sets.

It can be seen from Figure 15 that as the amount of data in the dataset increases, the authentication accuracy of the trained network increases. When the data volume is 1000, the authentication accuracy of the trained model can reach more than 90%. When the data volume is 3000, the accuracy reaches more than 96%. It can be found that the proposed authentication model can achieve a high classification accuracy even when the data volume is small. When the data volume is small, increasing the data volume in the training set can improve the authentication accuracy significantly. However, when the data volume reaches 4000, with the increase of data volume, the rate of accuracy improvement will be significantly reduced. We can see that, compared with the model trained with 4000 data volume, the improvement of the accuracy of the model trained with 8000 data volume is only 1%.

**Figure 16:**
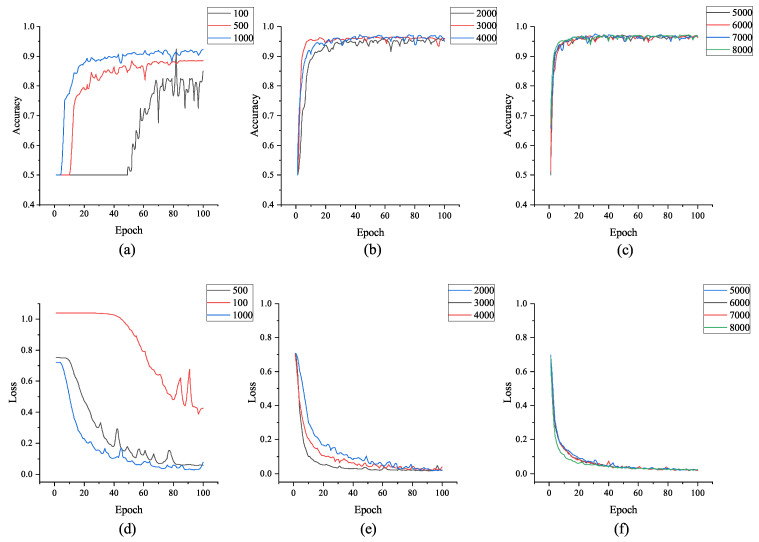
Accuracy curves and loss curves achieved by the model on the validation set after training with different numbers of data sets: (**a**–**c**) the accuracy curves; (**d**–**f**) the loss curves.

Except for the accuracy requirement, a good model also needs to have good convergence and robustness. Figure 16 shows the training accuracy and training loss curves of the model during the training process using various data sets with different sizes. It can reflect the convergence and robustness of the model.

From Figure 16, we can see that when the amount of data is relative small (less than 1000), although the model can eventually achieve good accuracy (about 90%), it takes lots of iterations and high volatility to reach convergence points. However, when the amount of data reaches 3000, the convergence speed increases significantly and it is able to maintain a stable state at a high accuracy. Therefore, we can see form the results that the proposed authentication model can achieve a high accuracy and stability when the data volume is low. Our model is more suitable for implicit authentication tasks in mobile devices since the battery and computing power is limited to support the collection of large amounts of data. Meanwhile, it can be well adapted to the task of stepping-based implicit authentication.

### 4.3. Performance Comparison of Three Different Methods

We compare the authentication method proposed in this paper with the following two methods:

1. SVM: Support vector machines are a class of generalized linear classifiers that perform binary classification of data in a supervised learning fashion. A decision boundary is a maximum margin hyperplane solved for the learned samples. In the implicit gait-based authentication task using SVM, instead of converting the gait signal to an image, we directly intercept the gait signal in a time window of length 150 and then input the intercepted samples directly into the model for training.
2. CNN: In using CNN for gait-based implicit authentication, we convert the gait signal into an image and then input the image into the network for training altogether. The difference is that the CNN network uses two fully connected layers as the classifier of the network, while the CNN-LSTM network proposed in this paper uses two LSTM layers as the classifier of the model.

**Figure 17:**
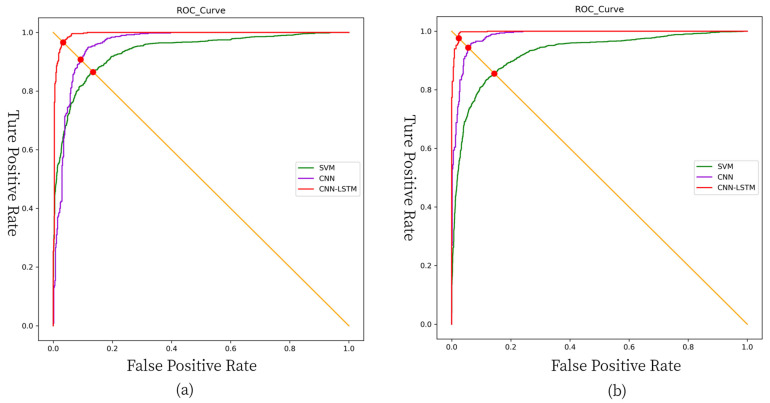
ROC curves: (**a**) the ROC curves of the three types of models when the number of training sets is 2000; (**b**) the ROC curves of the three types of models when the number of training sets is 8000.

We trained three models using two datasets with data amounts of 2000 and 8000, respectively, and the performance of the trained models on the validation set is shown in Table 9. The ROC curves for these models are shown in Figure 17.

Among these three types of models involved in the comparison, SVM has the worst authentication effect because the original gait signal is directly input into the SVM model and no feature selection is performed. In contrast, converting the original signal into a gait signal and feeding it directly into the deep learning model gives a better authentication result (regardless of whether the model is CNN or CNN+LSTM). This indicates that the method of converting the signal into an image and feeding it into the deep learning network can eliminate the manual feature selection step compared with the traditional machine learning method, and can perform feature extraction more easily and quickly automatically.

**Figure 18:**
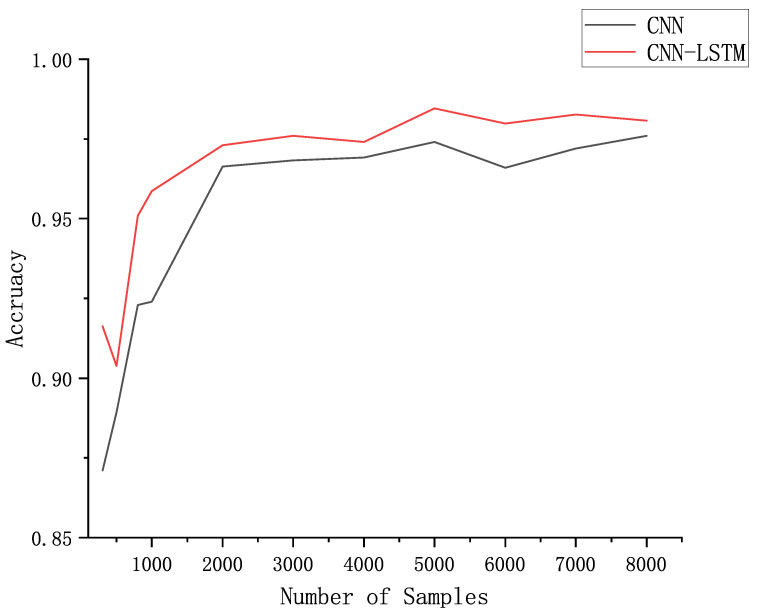
Accuracy of CNN models and CNN-LSTM models on datasets with different amounts of data.

We also compared the experimental results of CNN and CNN-LSTM models on different amounts of data, and the experimental results are shown in Figure 18.

The two curves in the figure are the accuracy curves of the CNN and CNN-LSTM models on the validation set obtained after the models were trained with different numbers of training sets. From the figure, it can be seen that the accuracy of CNN-LSTM is always higher than that of CNN model, especially when the amount of data in the training set is less than 1000, the accuracy of CNN-LSTM is much higher than that of CNN, which indicates that CNN-LSTM model achieves better recognition results when the data set is smaller. From the figure, the recognition accuracy of CNN-LSTM model is high but fluctuates more when the data volume is less than 1000, so the data volume of 2000 is more suitable for the classification recognition task in this paper.

The CNN+LSTM authentication method proposed in this paper has higher authentication accuracy as well as lower False Acceptance Rate(FAR) and False Rejection Rate(FRR) compared to CNN. This difference is more obvious again when the data volume is small, which indicates that the CNN+LSTM model has better performance and the model trained with a smaller data volume has higher authentication accuracy.

**Figure 19:**
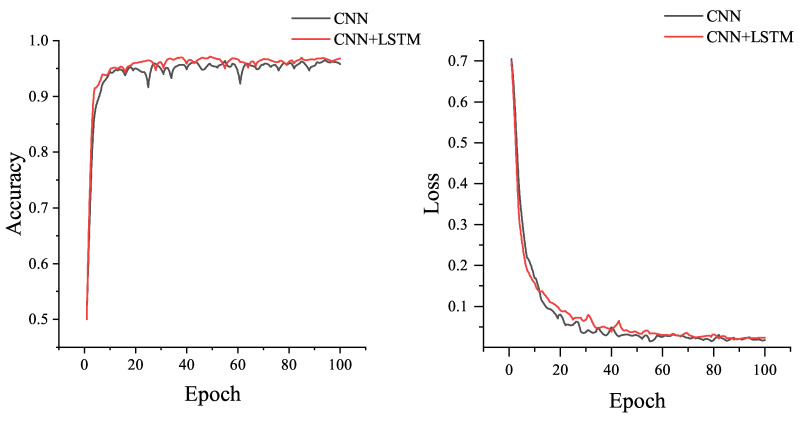
Training accuracy and loss curves of CNN and CNN-LSTM on small data size datasets.

The following figure shows the accuracy curves and training loss curves of CNN network and CNN+LSTM network during the training process when the amount of data is 2000. From Figure 19, we can see that the CNN+LSTM model can converge to a higher authentication accuracy more quickly in comparison.

### 4.4. Complexity Analysis

Based on the approach mentioned by [34], we performed a complexity analysis of the proposed deep learning model. They assume convolution is implemented as a sliding window and that the nonlinearity function is computed for free. For convolutional kernels, they use Formula (4) to compute the number of floating-point operations (FLOPs).

(4) $$\;\mathrm{FLOPs}\;=2HW\left(C_{in}K^{2}+1\right)C_{\mathrm{out}}$$

where *H*, *W* and $C_{in}$ are height, width and number of channels of the input feature map, *K* is the kernel width (assumed to be symmetric), and $C_{out}$ is the number of output channels.

For fully connected layers compute FLOPs as:

(5) FLOPs=(2I−1)O,

where *I* is the input dimensionality and *O* is the output dimensionality. The complexity of the model after calculation is shown in the Table 10.

### 4.5. Discussion

Our proposed EDIA algorithm achieves better classification results on the public dataset proposed by Matteo Gadaleta et al. [11], but still suffers from the following shortcomings (1) The dataset is small and insufficient to support the training of an authentication network with strong generalization capability, and we will subsequently collect more data using smartphones to expand the number of datasets. (2) Because of the limitation of cell phone security, we are unable to evaluate the impact on the energy consumption and memory of mobile devices after the model is deployed to them.

## 5. Conclusions

In this paper, we propose EDIA, an implicit authentication architecture, which enhances security of mobile devices via accuracy of user authentication on mobile devices. EDIA utilizes the gait data captured by build-in sensors as the unique behavioral characteristics to identify users. We design a hybrid deep learning model composed of a CNN and a LSTM in tandem for gait data modelling based on mobile device build-in sensors input collected in daily life. Our results have shown that a hybrid deep learning model (CNN + LSTM) increased the accuracy of gait model by more than 4% compared to a traditional machine learning SVM with simple CNN network structure. The proposed CNN + LSTM authentication method achieves the best authentication results when the structure contains two LSTM layers and the number of LSTM units is 100. To extract the main gait features and reduce noise at the same time, the original gait signal in one-dimension domain is converted into an image as the input of our deep learning model. In our architecture, the cloud server is responsible for generating the authentication model, while the edge node is employed to authenticating users in real time according to the model. We perform comprehensive experiments to evaluate the performance of EDIA, and the results show that: (i) our system outperforms other machine learning models by achieving an authentication accuracy higher than 97%; (ii) the model also has the great advantage of high authentication accuracy with small data sets, which makes it more suitable for mobile devices with limited energy and storage. For future work, we will study passive authentication using gait biometrics across mobile devices. In order to extend the applicability of the EDIA certification mechanism, we intend to further optimize the algorithmic model by using migration learning with unsupervised learning related methods. This will allow EDIA to be applied to multiple different mobile devices of the same user and optimize the authentication process when switching between devices for the same user.

## Figures and Tables

**Table 1:** Limitations of different behavioral biometric methods for mobile devices implicit authentication.

Behavioral Biometric Approach	Limitations
Touchscreen Interactions	(1) Requires the users to actively interact with the device’s touchscreen for authentication
	(2) Holding the phone in a different orientation makes a big difference in the way the user touches the phone
	(3) Users interact with the touch screen in different activity states such as walking, running, standing, sitting, etc. in very different ways
Behavioral Profiling	(1) Users’ moods and emotions affect the way they interact with different applications and services
	(2) Behavioral profiling data are difficult to access
Keystroke Dynamics	(1) Requires the user to actively interact with the device’s keyboard for authentication
	(2) User authentication is only possible when the user types on the keyboard
Hand waving Patterns	(1) Requires the user to actively interact with the device
	(2) Need special hand waving patterns to authenticate users
	(3) Multiple users may have the same hand waving patterns

**Table 2:** Key gait-based implicit authentication methods for mobile devices.

Research	Performance
Mäntyjärvi et al. [20]	EER 7%
Thang et al. [21]	Accuracy 79.1% (in time domain)
	Accuracy 92.7% (in frequency domain)
Muaaz et al. [22]	EER 22.49–33.30%
Nickel et al. [23]	FNMR:10.42%/FMR: 10.29%
Zhong et al. [24]	EER2.88% 7.22%
Damaševicius et al. [25]	EER 5.7%
Kašys et al. [26]	Accuracy 97% (correct identification)
	F-score 94%
Xu et al. [27]	Recognition Accuracy 32%; EER 3.5%
Abo El-Soud et al. [28]	Accuracy 97.8%; ERR 1.04%
	FAR 2.03%; FRR 0.04%
Papavasileiou et al. [29]	EER 0.01% 0.16%;
	FAR 0.54% 1.96%

**Table 3:** Parameters of each layer of CNN network.

Layer Name	Kernel Size	Kernel Num	Padding	Stride
Conv1	11	48	2	4
Maxpooling1	3	None	0	2
Conv2	5	128	2	1
Maxpooling2	3	None	0	2
Conv3	3	192	1	1

**Table 4:** The number of gait data for each user.

User ID	Number	User ID	Number	User ID	Number	User ID	Number
u001	572	u026	607	u014	264	u039	736
u002	2653	u027	692	u015	200	u040	1221
u003	1478	u028	2937	u016	875	u041	384
u004	1014	u029	1327	u017	328	u042	510
u005	195	u030	556	u018	8889	u043	1228
u006	376	u031	799	u019	977	u044	240
u007	1165	u032	141	u020	1014	u045	196
u008	380	u033	2863	u021	637	u046	1174
u009	270	u034	1176	u022	193	u047	422
u010	907	u035	1097	u023	6064	u048	130
u011	588	u036	1051	u024	2569	u049	304
u012	241	u037	299	u025	645	u050	190
u013	382	u038	449				

**Table 5:** The authentication performance of the model on these 50 datasets.

	User 1	User 2	User 3	User 4	User 5	User 6	User 7	User 8	User 9	User 10
**Accuracy**	0.974	0.979	0.984	0.984	0.979	1	0.982	1	0.989	0.984
**FRR**	0.039	0.013	0.010	0.020	0	0	0.020	0	0.020	0.029
**FAR**	0.010	0.026	0.019	0.010	0.038	0	0.015	0	0	0
**EER**	0.960	0.973	0.980	0.985	0.980	1	0.980	1	1	0.990
	**User 11**	**User 12**	**User 13**	**User 14**	**User 15**	**User 16**	**User 17**	**User 18**	**User 19**	**User 20**
**Accuracy**	0.974	0.979	0.949	0.979	0.979	0.974	0.959	0.984	0.982	0.987
**FRR**	0.003	0	0.076	0.020	0	0.010	0	0.004	0.020	0.010
**FAR**	0.010	0.038	0.021	0.020	0.038	0.038	0.074	0.025	0.015	0.014
**EER**	0.990	0.980	0.940	0.980	0.960	0.970	0.980	0.980	0.980	0.980
	**User 21**	**User 22**	**User 23**	**User 24**	**User 25**	**User 26**	**User 27**	**User 28**	**User 29**	**User 30**
**Accuracy**	0.989	0.979	0.995	0.937	0.989	0.979	0.934	0.956	0.989	0.974
**FRR**	0.010	0	0.002	0.052	0.010	0	0,070	0.017	0.005	0.030
**FAR**	0.010	0.038	0.005	0.071	0.01	0.038	0.060	0.066	0.014	0.022
**EER**	0.990	0.980	0.996	0.926	0.990	0.980	0.930	0.936	0.985	0.980
	**User 31**	**User 32**	**User 33**	**User 34**	**User 35**	**User 36**	**User 37**	**User 38**	**User 39**	**User 40**
**Accuracy**	0.969	0.898	0.959	0.989	0.977	0.979	0.939	0.959	0.984	0.952
**FRR**	0.020	0.145	0.027	0.005	0.025	0.034	0.042	0.075	0.010	0.041
**FAR**	0.039	0.045	0.051	0.014	0.020	0.005	0.076	0	0.019	0.0546
**EER**	0.960	0.840	0.956	0.980	0.975	0.985	0.940	0.940	0.980	0.945
	**User 41**	**User 42**	**User 43**	**User 44**	**User 45**	**User 46**	**User 47**	**User 48**	**User 49**	**User 50**
**Accuracy**	0.959	0.994	0.987	0.929	1	0.989	0.949	0.838	1	0.949
**FRR**	0.058	0.010	0.010	0.096	0	0.005	0.076	0.210	0	0.041
**FAR**	0.020	0	0.014	0.042	0	0.014	0.021	0.095	0	0.058
**EER**	0.980	1	0.990	0.940	1	0.990	0.960	0.80	1	0.940

**Table 6:** The authentication performance of the model on these 50 datasets.

Accruacy	<90%	90–97%	97–99%	100%
**Number**	2	14	30	4

**Table 7:** Precision results of different neural network.

Model ID	Number of LSTM Layers	Memory Units in LSTM Layer	Accuracy	Training Time/(seconds)
1	1	50	0.969	667.246
2	1	100	0.972	680.387
3	1	150	0.964	713.444
4	2	150 and 50	0.975	926.419
5	2	150 and 100	0.977	927.310
6	2	150 and 150	0.972	934.193

**Table 8:** Precision results of different neural network.

Num	100	500	1000	2000	3000	4000	5000	6000	7000	8000
Accuracy	0.760	0.907	0.932	0.950	0.960	0.967	0.969	0.974	0.973	0.977

**Table 9:** Certification effect of the three methods after training on different numbers of datasets.

	2000			8000				
	Accuracy	FRR	FAR	EER	Accuracy	FRR	FAR	EER
SVM	0.783	0.23	0.31	0.864	0.862	0.14	0.15	0.855
CNN	0.883	0.153	0.053	0.908	0.942	0.032	0.034	0.944
CNN+LSTM	0.969	0.026	0.032	0.966	0.977	0.021	0.0274	0.976

**Table 10:** Certification effect of the three methods after training on different number of datasets.

Complexity	Number
Total params	346,210
Total Memory	2.31 M
Total Flops	130.35 MFlops
Total MenR + W	5.42 MB

## Data Availability

Restrictions apply to the availability of these data. Data was obtained from Gadaleta et al. [11] and are available http://signet.dei.unipd.it/research/human-sensing/ with the permission of Gadaleta et al.

## References

[1] Kim Y., Oh T., Kim J. (2015). Analyzing user awareness of privacy data leak in mobile applications. Mob. Inf. Syst..

[2] Li Y., Xue F., Fan X., Qu Z., Zhou G. (2018). Pedestrian walking safety system based on smartphone built-in sensors. IET Commun..

[3] Li Y., Li X. (2016). Chaotic hash function based on circular shifts with variable parameters. Chaos Solitons Fractals.

[4] Patel V.M., Chellappa R., Chandra D., Barbello B. (2016). Continuous User Authentication on Mobile Devices: Recent progress and remaining challenges. IEEE Signal Process. Mag..

[5] De Luca A., Hang A., Brudy F., Lindner C., Hussmann H. Touch me once and i know it’s you! implicit authentication based on touch screen patterns. Proceedings of the SIGCHI Conference on Human Factors in Computing Systems.

[6] Jakobsson M., Shi E., Golle P., Chow R. Implicit authentication for mobile devices. Proceedings of the 4th USENIX Conference on Hot Topics in Security, USENIX Association.

[7] Muaaz M., Mayrhofer R. (2017). Smartphone-Based Gait Recognition: From Authentication to Imitation. IEEE Trans. Mob. Comput..

[8] Peinado-Contreras A., Munoz-Organero M. (2020). Gait-Based Identification Using Deep Recurrent Neural Networks and Acceleration Patterns. Sensors.

[9] Shiraga K., Makihara Y., Muramatsu D., Echigo T., Yagi Y. GEINet: View-invariant gait recognition using a convolutional neural network. Proceedings of the 2016 International Conference on Biometrics (ICB).

[10] Cao S., Wen L., Li X., Gao L. Application of Generative Adversarial Networks for Intelligent Fault Diagnosis. Proceedings of the 2018 IEEE 14th International Conference on Automation Science and Engineering (CASE).

[11] Gadaleta M., Rossi M. (2016). IDNet: Smartphone-based Gait Recognition with Convolutional Neural Networks. Pattern Recognit..

[12] Frank M., Biedert R., Ma E., Martinovic I., Song D. (2013). Touchalytics: On the Applicability of Touchscreen Input as a Behavioral Biometric for Continuous Authentication. IEEE Trans. Inf. Forensics Secur..

[13] Li F., Clarke N., Papadaki M., Dowland P. Behaviour Profiling for Transparent Authentication for Mobile Devices. Proceedings of the 10th European Conference on Information Warfare and Security 2011 (ECIW).

[14] Li F., Clarke N., Papadaki M., Dowland P. (2014). Active authentication for mobile devices utilising behaviour profiling. Int. J. Inf. Secur..

[15] Bassu D., Cochinwala M., Jain A. A new mobile biometric based upon usage context. Proceedings of the 2013 IEEE International Conference on Technologies for Homeland Security (HST).

[16] Eagle N., Pentland A.S. (2006). Reality mining: Sensing complex social systems. Pers. Ubiquitous Comput..

[17] Lee H., Hwang J.Y., Lee S., Kim D.I., Lee S.H., Lee J., Shin J.S. (2019). A parameterized model to select discriminating features on keystroke dynamics authentication on smartphones. Pervasive Mob. Comput..

[18] Peng G., Zhou G., Nguyen D.T., Qi X., Yang Q., Wang S. (2016). Continuous authentication with touch behavioral biometrics and voice on wearable glasses. IEEE Trans. Hum. Mach. Syst..

[19] Yang L., Guo Y., Ding X., Han J., Liu Y., Wang C., Hu C. (2015). Unlocking Smart Phone through Handwaving Biometrics. IEEE Trans. Mob. Comput..

[20] Mantyjarvi J., Lindholm M., Vildjiounaite E., Makela S., Ailisto H.A. Identifying users of portable devices from gait pattern with accelerometers. Proceedings of the (ICASSP’05), IEEE International Conference on Acoustics, Speech, and Signal Processing.

[21] Thang H.M., Viet V.Q., Thuc N.D., Choi D. Gait identification using accelerometer on mobile phone. Proceedings of the 2012 International Conference on Control, Automation and Information Sciences (ICCAIS).

[22] Muaaz M., Mayrhofer R. An analysis of different approaches to gait recognition using cell phone based accelerometers. Proceedings of the International Conference on Advances in Mobile Computing & Multimedia.

[23] Nickel C., Busch C., Rangarajan S., Möbius M. Using hidden markov models for accelerometer-based biometric gait recognition. Proceedings of the 2011 IEEE 7th International Colloquium on Signal Processing and its Applications.

[24] Zhong Y., Deng Y., Meltzner G. Pace independent mobile gait biometrics. Proceedings of the 2015 IEEE 7th International Conference on Biometrics Theory, Applications and Systems (BTAS).

[25] Giorgi G., Saracino A., Martinelli F. (2021). Using recurrent neural networks for continuous authentication through gait analysis. Pattern Recognit. Lett..

[26] Kašys K., Dundulis A., Vasiljevas M., Maskeliūnas R., Damaševičius R. (2020). BodyLock: Human Identity Recogniser App from Walking Activity Data. Lecture Notes in Computer Science, Proceedings of the International Conference on Computational Science and Its Applications, Cagliari, Italy, 1–4 July 2020.

[27] Xu W., Shen Y., Luo C., Li J., Li W., Zomaya A.Y. (2020). Gait-Watch: A Gait-based context-aware authentication system for smart watch via sparse coding. Ad Hoc Netw..

[28] El-Soud M.W.A., Gaber T., AlFayez F., Eltoukhy M.M. (2021). Implicit authentication method for smartphone users based on rank aggregation and random forest. Alex. Eng. J..

[29] Papavasileiou I., Qiao Z., Zhang C., Zhang W., Bi J., Han S. (2021). GaitCode: Gait-based continuous authentication using multimodal learning and wearable sensors. Smart Health.

[30] Hinton G.E., Osindero S., Teh Y.W. (2006). A fast learning algorithm for deep belief nets. Neural Comput..

[31] Ji S., Xu W., Yang M., Yu K. (2012). 3D convolutional neural networks for human action recognition. IEEE Trans. Pattern Anal. Mach. Intell..

[32] Karpathy A., Toderici G., Shetty S., Leung T., Sukthankar R., Fei-Fei L. Large-scale video classification with convolutional neural networks. Proceedings of the IEEE Conference on Computer Vision and Pattern Recognition.

[33] Taigman Y., Yang M., Ranzato M., Wolf L. Closing the gap to human-level performance in face verification. deepface. Proceedings of the IEEE Computer Vision and Pattern Recognition (CVPR).

[34] Molchanov P., Tyree S., Karras T., Aila T., Kautz J. Pruning Convolutional Neural Networks for Resource Efficient Inference. Proceedings of the 5th International Conference on Learning Representations, ICLR.

